# Genomic insights into the emergence and spread of antimicrobial-resistant bacterial pathogens

**DOI:** 10.1126/science.aar3777

**Published:** 2018-05-18

**Authors:** Stephen Baker, Nicholas Thomson, François-Xavier Weill, Kathryn E. Holt

**Affiliations:** 1 Oxford University Clinical Research Unit, Ho Chi Minh City, Vietnam; 2 Centre for Tropical Medicine and Global Health, Oxford University, Oxford, UK; 3 The Department of Medicine, University of Cambridge, Cambridge, UK; 4 The Wellcome Trust Sanger Institute, Cambridge, UK; 5 The London School of Hygiene and Tropical Medicine, London, UK; 6 Institut Pasteur, Paris, France; 7 Department of Biochemistry and Molecular Biology, Bio21 Molecular Science and Biotechnology Institute, University of Melbourne, Parkville, Victoria, Australia

## Abstract

Whole-genome sequencing (WGS) has been vital for revealing the rapid temporal and spatial evolution of antimicrobial resistance (AMR) in bacterial pathogens. Some antimicrobialresistant pathogens have outpaced us, with untreatable infections appearing in hospitals and the community. However,WGS has additionally provided us with enough knowledge to initiate countermeasures. Although we cannot stop bacterial adaptation, the predictability of many evolutionary processes in AMR bacteria offers us an opportunity to channel them using new control strategies. Furthermore, by usingWGS for coordinating surveillance and to create a more fundamental understanding of the outcome of antimicrobial treatment and AMR mechanisms, we can use current and future antimicrobials more effectively and aim to extend their longevity.

When antimicrobial drugs were introduced into clinical usage in the mid- 20th century, they had an astonishing impact on human health. Infectious bacteria that had threatened our survival were now at the mercy of a chemical arsenal. Previously fatal infections, from whooping cough and scarlet fever to tuberculosis and syphilis, were no longer considered a threat. Antimicrobials substantially reduced the risks associated with child birth, injuries, and invasive medical procedures. What has followed in the subsequent 70 years or so has been an uncontrolled microbiological experiment conducted on an unprecedented scale. Initially we identified a plethora of new antimicrobial classes targeting different essential bacterial functions, but we deployed them haphazardly in ever-increasing quantities. Now antimicrobial resistance (AMR) poses a genuine threat to human health, with the potential to return us to a situation where common infections are as untreatable as they were in the pre-antimicrobial era (*1*).

Humans did not create AMR; we simply promoted it by applying evolutionary pressure. Almost all antimicrobials have chemical similarities with compounds that can be found naturally; AMR genes have been found deep in the permafrost (*2*) and arose long before humankind’s ability to synthesize antibacterial chemicals and use them en masse. Therefore, AMR in bacterial populations is a largely predictable phenomenon; the more commonly a specific antimicrobial compound is used, the more likely it is that resistance will emerge and be maintained in an exposed microbial population. The specific dynamics of the processes associated with AMR are, however, less predictable. The rapidity with which diverse AMR phenotypes have emerged and become established within human, animal, and wider environmental populations of microbes has been astonishing and most likely accelerated by concurrent advances in human development, mobilization, and population growth.

***“The first reports of penicillin-resistant infections occurred early in the 1940s, but a penicillinase was described even before the continued clinical usage of the prototype antibiotic.”***

## The evolutionary dynamics of antimicrobial resistance

How resistance is maintained and distributed within bacterial populations is a function of the organism’s lifestyle (i.e., transmission mode, colonization, and pathogenicity) and the genetic basis for resistance, which can be either intrinsic (i.e., the organism naturally lacks the specific pathway targeted by the drug), mutation associated (i.e., induced changes are passed vertically to descendants), or acquired via horizontal gene transfer (HGT) between organisms (with acquired genes then being passed vertically to progeny). The first reports of penicillin-resistant infections occurred early in the 1940s, but a penicillinase was described even before the continued clinical usage of the prototype antibiotic (*3*). Since then, there have been numerous examples of the rapid emergence of bacteria exhibiting resistance to a specific antimicrobial class soon after its introduction (*4*). However, in the past decade, through the advent of high-throughput wholegenome sequencing (WGS), we have been able to make substantive advances in understanding the dynamics of AMR evolution and spread in bacterial populations.

**Fig. 1:**
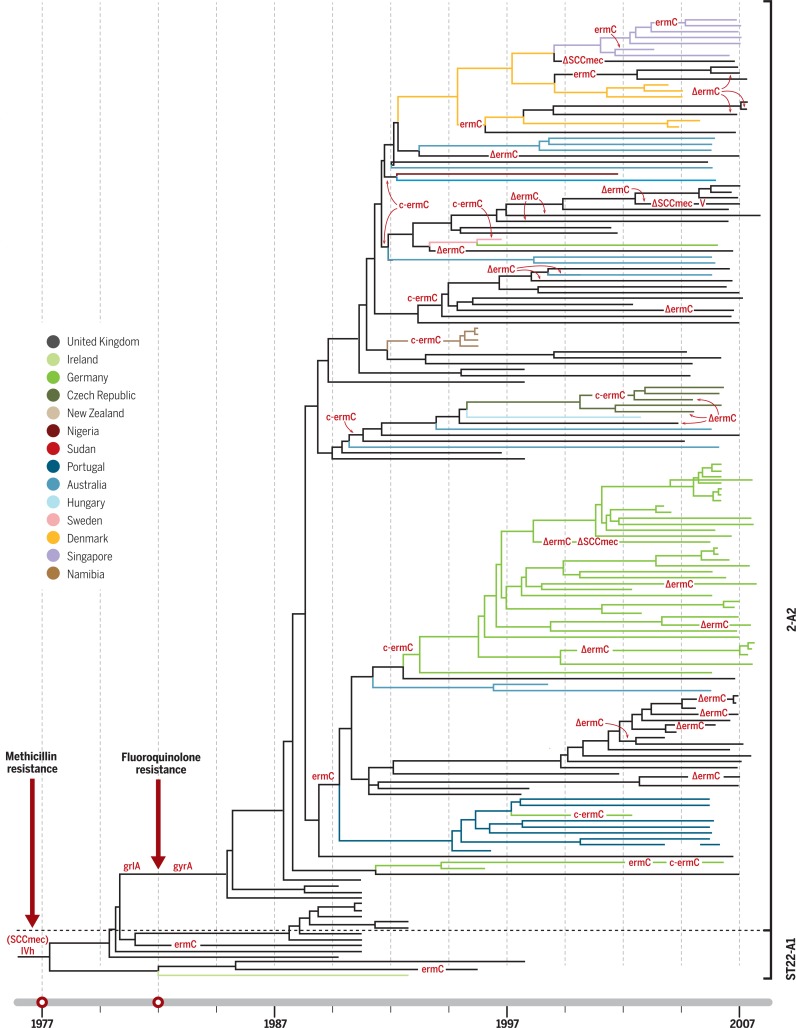
**The time line of the ST22 MRSA pandemic**. Bayesian phylogenetic tree reconstructing the ST22 MRSA pandemic over a 30-year period (*8*). Maximum clade credibility tree of ST22 MRSA based on BEAST analysis using a variable clock rate (uncorrelated lognormal) model. Tips of the tree are constrained by isolation dates; the time scale is shown at the base of the tree. Gains and losses (D) of genetic determinants for resistance to methicillin (SCC*mec*), fluoroquinolones (point mutations in *grlA* and *gyrA*), erythromycin (plasmid-encoded *ermC*), and clindamycin (mutations in ermC leader peptide region, c-*ermC*) have been mapped on the tree by applying the parsimony criterion. The figure depicts two pivotal events: the acquisition of methicillin resistance around 1977 and fluoroquinolone resistance in 1982 (red arrows). This clone then underwent rapid international spread, including countryspecific clonal expansions; countries highlighted in color.

WGS has become the key technology for understanding pathogen evolution, population dynamics, and genomic epidemiology, as it provides a far greater degree of reproducibility, standardization, and resolution than previous genotyping methods (*5*). By capturing both the neutral evolution of the population— for tracking transmission and diversification of the organism—and the genetic determinants of AMR, WGS can reveal detailed temporal and spatial dynamics of AMR evolution and simultaneously infer the impact of AMR selection on pathogen populations. Much of the pioneering WGS-based AMR work was focused on the opportunistic Gram-positive human pathogen *Staphylococcus aureus*, particularly with respect to the emergence of methicillin resistance (MRSA) in health care facilities in Europe (*6*). MRSA is still among the best examples of how AMR variants can rapidly emerge, be efficiently maintained, and spread at different spatiotemporal scales, ranging from individual hospital wards to health care networks, and internationally within human populations (Fig. 1). MRSA was first observed in 1960, within a year of the introduction of secondgeneration b-lactams, such as methicillin, into clinical practice. However, phylogenetic reconstruction showed that MRSA actually emerged in the 1940s via HGT of the staphylococcal cassette chromosome *mec* (SCC*mec*) element, as a consequence of the initial mass usage of penicillin (7). WGS data shows that MRSA has arisen on numerous occasions independently in different subpopulations on different continents (e.g., USA300, ST22 in Europe, and ST93 in Australia) through parallel HGT events and spread throughout health care systems (*6*). The history of health care–associated MRSA in the later part of the 20th century was punctuated by frequent epidemics associated with highly successful clones, such as EMRSA-15 (ST22), which was first described in the United Kingdom in the 1990s and then spread throughout Europe, and then intercontinentally (Fig. 1) (*8*). Notably, a fluoroquinolone-resistant EMRSA-15 variant arose in the United Kingdom soon after clinical trials with ciprofloxacin in the 1980s, with point mutations in the DNA gyrase and topoisomerase IV genes. This critical event was the apparent trigger for the subsequent pandemic spread of a fluoroquinolone-resistant MRSA variant (Fig. 1) (*8*).

## The global dissemination of antimicrobial-resistant clones

MRSA epitomizes a now all-too-familiar evolutionary route by which successful AMR clones emerge in response to local antimicrobial usage, undergo population expansion under selection from sustained antimicrobial exposure, and then explode into pandemic spread. The finer details are organism specific and dependent on their particular evolutionary landscape (e.g., mechanisms of resistance, fitness costs, modes of transmission, and host range), but all follow a similar basic trajectory, mirroring that observed in the recent MEGA-plate experiment (*9*). Briefly, exposure of susceptible bacteria to antimicrobial drugs will result in the local emergence of resistant mutants. This happens continuously, as a genetically diverse pool of pathogens are exposed to a range of different compounds at different concentrations. Most resistant mutants will be purged quickly from the population, either through genetic drift or because they are less fit for onward transmission. For example, WGS data have shown that that a few common resistance mutations emerge repeatedly in *Mycobacterium tuberculosis* during the treatment of individuals but that these are rarely transmitted (*10*). However, occasionally a resistant mutant will have a sufficient fitness advantage to undergo local clonal expansion in a subset of infections. This occurs through a combination of ongoing antimicrobial exposures and/or a genetic background that moderates the fitness cost, e.g., the compensatory mutations in rifampicin-resistant *M. tuberculosis* (*11*); the increased replication rate of *Salmonella* Typhi with fluoroquinolone resistance–associated DNA gyrase mutations (*12*); or chromosomal variants that ameliorate the cost of AMR plasmid carriage (*13*). Once established, the locally successful AMR clonemay face opportunities for further expansion, including potentially broader geographical dissemination and/or spillover into other host populations, depending on the mode of transmission and the extent of antimicrobial selection it encounters.

WGS investigations show that clonal expansion and ensuing geographical dissemination of pathogens can mostly be traced to the acquisition of a specific AMR determinant(s) like SCC*mec* in MRSA. This suggests the AMR element(s) function as the “king maker” within the various pathogen populations, determining which clones dominate locally, regionally, and globally. Some mobile AMR genes have played this role in multiple organisms and clones; e.g., CTX-M-15 has driven the success of *Escherichia* coli ST131 and several *Klebsiella pneumoniae* clones (CG14/15, ST101) (*14*, *15*). Equally, AMR genes also benefit by association with certain plasmid vectors or host bacterial clones, which act as vehicles for dissemination. *K. pneumoniae* is host to several key mobile AMR genes and has played a pivotal role in the global dissemination of various extended spectrum b-lactamases (ESBLs) and the carbapenemases KPC and NDM-1 (*15*). This association may be linked to *K. pneumoniae’s* broad ecological range and propensity for HGT, which provide a conduit for AMR gene trafficking from a very large gene pool into the smaller subpopulations of human-associated bacteria.

Another common reoccurring observation is the accumulation of additional resistance mechanisms in an already established AMR clone, such as fluoroquinolone resistance in EMRSA-15 (*8*). This phenomenon is likely driven by escalating antimicrobial use to tackle AMR infections, accompanied by a relaxation of selective constraints and an increased effective population size of the successful clone. It is particularly common in organisms that can accumulate multiple AMR genes through HGT, particularly within the Enterobacteriaceae (*14*, *15*), but is also evident in the highly clonal and evolutionarily constrained *M. tuberculosis*, in which resistance to isoniazid via a mutation in *katG* commonly precedes further AMR mutations (*10*).

## Health care–associated “superbugs”

AMR organisms are highly destructive in hospitals. Modern medicine relies on antimicrobial therapy and prophylaxis to protect against opportunistic infections, which affect approximately 1 in 10 hospitalized patients globally. In industrialized countries, health care–associated infections account for the vast majority of the communicable disease burden (*16*), but hospitals on all continents are now plagued by AMR infections. The combination of intensive antimicrobial exposure in hosts whose immune systems are struggling to defend against infecting bacteria can rapidly select for resistance. Several WGS studies have documented local emergence of resistance in hospitalized patients in response to specific antimicrobial exposures, which have been studied in individual infections, treatment episodes (*17*), and at the ward level (*18*). These studies show that many of the same mutational events arise repeatedly in different patients and in different host backgrounds, demonstrating that the emergence of AMR in many organisms within health care facilities is often predictable. Examples include the repeated acquisition of SCC*mec* (methicillin resistance), *walKR* mutations (vancomycin resistance) in *S*. *aureus* (*18*), and *lpx* disruptions (colistin resistance) in *Acinetobacter baumannii* (*17*).

**Fig. 2:**
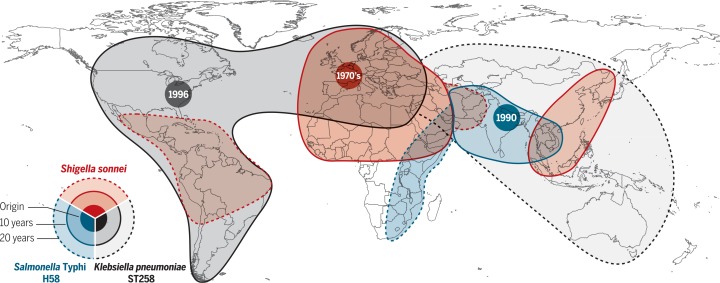
**Origin and blast radius for the clonal expansion for three multidrug-resistant Gram-negative bacteria clones.** The map summarizes data for the global dissemination of: dysentery causing *Shigella* sonnei clone lineage III-global, with a chromosomal insertion of a mobile genetic element encoding resistance to streptomycin, trimethoprim-sulfamethoxazole, and tetracycline (red); the typhoid fever pathogen *Salmonella* Typhi, clone H58, with a plasmid encoding resistance to chloramphenicol, ampicillin, trimethoprim-sulfamethoxazole, streptomycin, and tetracycline (blue); health care–associated *Klebsiella pneumoniae* clone ST258, carrying the KPC carbapenemase encoding resistance to all β-lactam antimicrobials, including carbapenems and third-generation cephalosporins (gray).

Although AMR organisms arise continuously, national- and international-level WGS snapshots show that most AMR infections are attributable to a few clones within the broad population of the specific pathogen. Thus, only a small fraction of emergent AMR variants is sufficiently fit for broader dissemination. WGS investigations of Gram-negative opportunistic pathogens mimic the pattern of MRSA, with clonal spread that begins as localized expansions, rapidly progressing to intercontinental spread (within years) and even global dissemination (within decades). Particularly concerning is *K*. *pneumoniae* clone ST258, which carries the plasmid-borne *K. pneumoniae* carbapenemase gene KPC that confers resistance to all b-lactams, including carbapenems and cephalosporins (*15*). KPC ST258 arose in the United States, where it began causing hospital outbreaks around 2005. After first spreading to Israel, by 2009, KPC ST258 was endemic in Greece and Italy and has since spread across Europe and South America and into Asia and Australia (Fig. 2). The arrival of the clone in new locations is linked to patients with a history of recent international travel to KPC ST258–endemic areas. Other carbapenemaseproducing *K. pneumoniae* clones have also emerged (e.g., OXA-48 ST405 in Spain and KPC ST11 in China), but these have remained relatively localized. Why a combination of the KPC gene in the ST258 *K. pneumoniae* host background has been so successful remains an important unanswered question.

Other relevant Gram-negative health care–associatedAMR clones include the ESBL-producing *E. coli* ST131, whose global dissemination has been so rapid that its initial geographical origins were obscured (*14*). *A. baumannii* Global Clone 1 (GC1) is probably the oldest multidrugresistant (MDR) hospital clone of *A. baumannii* and emerged in the 1980s after acquisition of a genomic island conferring resistance to all firstline antimicrobials. GC1 latterly accumulated resistance against fluoroquinolones and carbapenems (*19*). The prevention and management of infections with these highly resistant clones is a major health care challenge, and alternative strategies, such as vaccines and targeted immunotherapies, are urgently needed. However, *K. pneumoniae* KPC ST258, *E. coli* ST131, and *A. baumannii* GC1 all display extensive surface antigen diversification, complicating such approaches (*15*, *19*).

## Antimicrobial resistance in community-acquired infections

AMR is not only a substantial problem in health care systems but is also prevalent among a wide range of pathogens associated with communityacquired infections. WGS studies show that AMR in the community setting, as in hospitals, is similarly dominated by a small number of globally disseminated clones that have accumulated AMR determinants over time. The waterborne enteric diseases typhoid fever and dysentery provide two salient examples. The vast majority of MDR typhoid fever cases globally are caused by the *Salmonella* Typhi H58 clone, which emerged in South Asia in the early 1990s in association with an MDR plasmid and has since spread throughout Asia and into East Africa, accumulating fluoroquinolone resistance mutations in the genes encoding DNA gyrase and topoisomerase IV (Fig. 2) (*20*). Most pediatric cases of MDR shigellosis are caused by a *Shigella sonnei* clone that carries a mobile genetic element conferring resistance to almost all first-line drugs on its chromosome. The clone emerged in the 1970s and is now globally disseminated (Fig. 2) (*21*), with the same fluoroquinolone resistance mutations as in *S*. Typhi arising subsequently and spreading out from South Asia (*22*).

Sexually transmitted infections (STIs) present particular complications for understanding AMR in community-acquired disease, as their transmission is driven by complex human behavior. AMR in STIs share the same general evolutionary characteristics as AMR in health care–acquired infections, but have distinct transmission, diagnosis, and treatment parameters that result in distinct spatiotemporal dynamics. AMR in STIs are a major concern; data from locations with good STI surveillance systems suggest a general upward trend in bacterial STI incidence disproportionately associated with specific communities (*23*). In 2014, men-who-have-sex-with-men (MSM) represented <2% of the London adult population; however, 28% of all new STIs were diagnosed in this community. More specifically, 69% of all new cases of gonorrhea diagnosed in London were in MSM, and the emergence of some AMR variants of *Neisseria gonorrhoeae* have been specifically linked to MSM communities (*23*). AMR in *N. gonorrhoeae* is such a potential problem that it has been acknowledged by the World Health Organization (WHO) as being a major threat to human health (*24*). MDR variants leave increasingly limited treatment options, and there is a very real prospect of widespread resistance to ceftriaxone, the last remaining option for empirical monotherapy. Indeed, there have already been isolated reports of *N*. *gonorrhoeae* that exhibit resistance to all current treatments (*24*). One of the first epidemiological studies exploiting WGS for *N. gonorrhoeae* aimed to understand how particular AMR phenotypes had emerged. This study showed that reduced susceptibility against third-generation cephalosporins in the United States between 2000 and 2014 was the consequence of the expansion of two particular clones arising within the MSM community that possessed the mosaic *penA* resistance allele (25).

For other STIs, the situation is less clear. Despite reports of mutations in *Chlamydiatrachomatis* conferring in vitro resistance against macrolides (the first-line treatment for chlamydia), there is no evidence for the stable maintenance of these mutations during human infection (*26*). Similarly, intramuscular injection with benzathine penicillin appears to remain generally effective for treating syphilis (*Treponema pallidum*). However, we are missing key epidemiological information on many STIs. In well-resourced clinical settings, there is a move away from microbiological culture as the “gold standard” for the diagnosis of bacterial STIs and increasing reliance on molecular testing (*24*). Although molecular tests have the advantage of being rapid and sensitive, they have the disadvantage of being destructive and do not screen for potential AMR phenotypes (*24*). This situation is exacerbated in resource-limited settings where any form of diagnostic testing is rare, which results in a substantial underreporting of STIs and almost no AMR or pathogen prevalence data (*24*).

***“Sexually transmitted infections present particular complications for understanding AMR in community-acquired disease, as their transmission is driven by complex human behavior.”***

Another issue complicating AMR detection in STIs is the challenge of individual case management. A lack of diagnostic testing imposes a reliance on empirical syndromic therapy, which can have undesired consequences for driving the emergence of new AMR-STIs because of undirected antibiotic treatment. *Shigella* spp. are fecal-oral pathogens with a notoriously low infectious dose and are adept at acquiring new functions via HGT. *Shigella* has emerged as an enteric STI with a capacity for global dissemination of AMR genotypes. *Shigella* outbreaks in MSM communities have been sporadically observed since the 1970s (27). An increase in MSM-associated dysentery has been reported recently in the United Kingdom with a *Shigella flexneri* resistant to azithromycin in individuals with no history of travel to countries with highly endemic *Shigella* (28). Azithromycin is not routinely used to treat dysentery in the United Kingdom, but is the front-line treatment for gonococcal urethritis, syphilis, and chlamydia. The emergence of this *S. flexneri* variant was linked to the acquisition of a conjugative plasmid carrying various macrolide resistance genes, which was likely driven by azithromycin treatment for other STIs. Transmission of organisms via oro-anal sex, coupled with HIV-associated immunodeficiency, multiple sexual partners, and greaterexposure to STIs alongside therapeutic antimicrobials, created the “perfect storm” for the emergence of this specific MSM-associated AMR lineage.

## Foodborne dissemination of antimicrobial resistance

Humans are exposed to animal sources of AMR genes and bacteria through the food chain. The need for a “one-health” (i.e., considering the span of humans, animals, and their environment) strategy for AMR and infectious disease for surveillance and containment across the different sectors is well recognized. Nontyphoidal *Salmonella* (NTS), which is among the most common pathogens of humans and animals, are key for understanding AMR dynamics from a one-health perspective. In 2004, the Infectious Disease Society of America (IDSA) issued a report that presented a plausible catastrophic scenario of a highly fatal epidemic of MDR-NTS, illustrating how virulent AMR strains could rapidly escalate into major foodborne outbreaks threatening our food security. Indeed, large foodborne NTS outbreaks have been observed in recent decades, and NTS exhibiting resistance to last-line antimicrobials are beginning to be isolated.

The continued occurrence of MDR *Salmonella* Typhimurium (one of the most common types of NTS) as a cause of human infection personifies the one-health aspect of AMR and also highlights repeating patterns of AMR evolution. Antimicrobials have been used to treat and prevent infections in livestock since their discovery but were also used as growth promoters from the 1950s. In the early part of the 1960s, an increasing number of *S*. Typhimurium with transferable MDR phenotypes began to be isolated in the United Kingdom, with the first outbreak of MDR *S*. Typhimurium (phage type 27) in humans reported in 1959. This outbreak affected 102 patients; ~5% of isolates were resistant to streptomycin, sulfonamides, and tetracycline (*29*). In 1963, *S*. Typhimurium phage type DT29 emergedin the UnitedKingdom following the adoption of intensive farming methods using antibiotics for the rearing of calves (*30*). Subsequently, in 1965, >1200 and >500 MDR *S*. Typhimurium were isolated from cattle and humans, respectively. A recent WGS NTS investigation revealed that the AMR gene cassettes present in these early U.K. *Salmonella* outbreaks differed fromthose in historical *Salmonella* outbreaks in France, despite geographic proximity (*31*). This observation suggests that the emergence of MDR *S*. Typhimurium was caused by the independent acquisition of multiple AMR determinants followed by country-specific clonal expansions.

***“…globalization of the food industry means that inappropriate antimicrobial use in one part of the world has implications even for countries with strong controls on their own usage.”***

Observations from the 1960s were repeated in the 1980s when *S*. Typhimurium phage type DT104 with a genomic island encoding resistance against ampicillin, chloramphenicol, streptomycin, sulfonamides, and tetracyclines emerged in U.K. cattle (*32*). This epidemic strain successively acquired resistance to quinolones and trimethoprim. Over the coming years, DT104 became widely distributed in cattle, poultry, pigs, and sheep and in 1996, >4000 human infections were associated with MDR DT104 in the United Kingdom. MDR DT104 spread internationally throughout the 1990s, particularly in continental Europe and North America, and became established in multiple domestic animal populations. By 2001, DT104 represented >50% of all *S*. Typhimurium isolates in Eastern Europe (*33*). Local and global transmission routes were reconstructed by WGS, and the role of this zoonotic pathogen in the spread of AMRthrough interspecies transmission was elucidated (*34*). These data may have cast doubt on the dominance of local animals in spreading MDR DT104 to humans, but importantly, they highlighted substantial gaps in our AMR surveillance. Notably, the general contribution of imported food in spreading AMR bacteria to humans remains poorly understood.

**Fig. 3:**
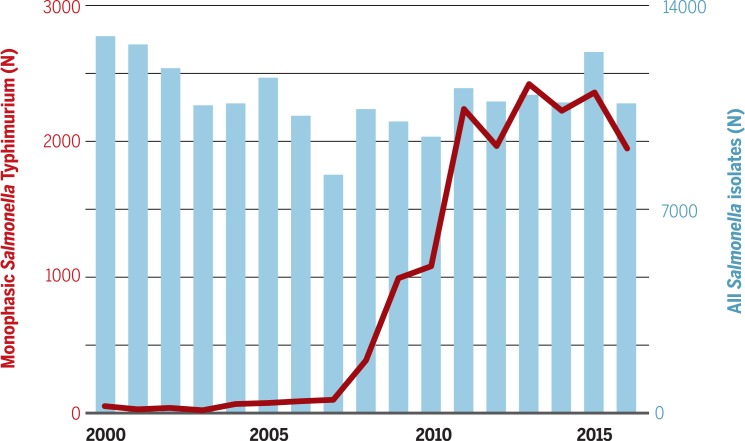
**The epidemic of monophasic Salmonella Typhimurium (1,4,[5],12:i:-).** The graph shows the number of *Salmonella* isolates from human infections at the French National Reference Centre for *Salmonella* during 2000 to 2016. The blue bars depict the total number of *Salmonella* spp. isolated by year over the defined period; the red plot depicts the number of *Salmonella* Typhimurium (1,4,[5],12:i:-) isolated by year.

The latest foodborne *S*. Typhimurium epidemic was associated with swine and attributed to a monophasic variant (1,4,[5],12:i:-), which emerged in Europe in the mid-2000s, as highlighted by spread of the clone in France from 2008 (Fig. 3) (*35*). Sequence data identified these organisms as one clone, despite belonging to multiple phage types, that was distinct from monophasic *S*. Typhimurium previously described in Spain and North America. These were found to have become MDR through the acquisition of a composite transposon, which replaced the flagella operon. These isolates had also acquired a genomic island, which encoded resistance to several heavy metals in pig-feed supplements. This European monophasic variant has now been reported in swine in the Midwestern United States, where it has become resistant to quinolones and third-generation cephalosporins (*36*).

It was proposed relatively early on that use of penicillins and tetracyclines in livestock was responsible for the emergence of MDR *S*. Typhimurium. This hypothesis was controversial, owing to the complexities of NTS epidemiology and the lack of molecular tools allowing high-resolution tracking of the incriminated bacteria in the different ecosystems. In the 1980s, epidemiology, combined with early molecular typing techniques, concluded that most AMR variants of NTS in the United States could be traced to animals (*37*). Antimicrobial use for growth promotion was banned by the European Union in 2006 and heavily regulated in the United States in 2017. However, globalization of the food industry means that inappropriate antimicrobial use in one part of the world has implications even for countries with strong controls on their own usage. The recent example of the worldwide dissemination of MDR *Salmonella* Kentucky ST198 via African poultry further highlights the requirement for global one-health approaches to tackle AMR (*38*).

## Staying one step ahead

It is indisputable that efforts to kill bacteria with chemicals will result in the selection, propagation, and dissemination of resistant variants. Data generated through WGS have revealed the rapid pace at which the bacteria can adapt to these chemicals. It is evident that some AMR pathogens have outpaced us, with untreatable infections appearing in hospitals and the community; but WGS studies have provided us with the tools and knowledge to initiate an intelligence-driven fightback. In particular, population genomics data at various spatiotemporal scales highlight many repeating patterns in the emergence and spread of AMR. The predictability of these evolutionary processes offers the opportunity to develop strategies to minimize the chance that new AMR clones are generated during individual treatment that will spread locally. For example, combination and sequential therapies may create conditions that constrain the fitness of emerging resistantmutants (*39*). These strategies are based broadly on the principle that adaptation to one class of antimicrobial drug may incur collateral sensitivity to another, such that their coordinated use imposes a roadblock to the emergence and spread of resistance. As diagnostics are generally lacking, the most practical option is likely to be empirical antimicrobial rotation as opposed to patient-tailored therapies. In theory, antimicrobial combinations or cycling can be employed at different levels (e.g., patients, wards, hospitals) and time scales (e.g., hours, days, months), depending on whether the goal is to limit the emergence of AMR within patients, or to confine the transmission of AMR variants. However, much work is required to determine the most effective way to restrict emergence and spread of differing resistance phenotypes in different settings (*40*). These approaches have the potential to lengthen the life of current antimicrobials and are vital for sustaining the efficacy of new antimicrobials as they are introduced.

A further important insight from WGS is that while resistance arises constantly during individual infections, most AMR variants represent a minimal risk with limited potential for transmission beyond the index patient. Hence, the major burden of AMR is associated with a few high-risk clones that spread easily and accumulate additional AMR phenotypes. It is these clones that represent the greatest risk beyond the individual patient and should be targeted more aggressively for containment. Work is still needed to understand the mechanisms underlying these apparently superfit AMR clones, and WGS studies will be vital for this process. Even in the absence of precise mechanisms,WGS can be deployed immediately for hospital infection control and public health surveillance to identify and target clones with epidemic potential as they arise.

The spatiotemporal dynamics of AMR evolution revealed by WGS studies clearly illustrate that microbial populations do not respect political boundaries; hence, it is imperative that AMR genomic surveillance data are combined internationally between different sectors in a one-health approach (i.e., across medical, veterinary, agricultural, and environmental settings). Such data sharing is essential to harness the power of genomic surveillance to identify and monitor evolutionary trends and population dynamics and to identify superfit AMR clones as they emerge and spread. The rapid pace of the global spread of AMR organisms, such as fluoroquinolone-resistant *Shigella* (*22*), indicates that these efforts have to be implemented in real time, as has been argued for the emergence of novel pathogens (*5*). This is the vision of the Global Microbial Identifier Project, the WHO, and other international bodies, but it has yet to gain international support from governments and industries.

AMR is a truly global health problem, one that we cannot ignore or attempt to counter with increasingly powerful antimicrobial agents. WGS has allowed us to understand the dynamics of AMR and the chaos we have created through haphazard antimicrobial usage. The data are stark. However, recognizing the complexity and assessing the magnitude of the task ahead is the first fundamental step in tackling the global AMR crisis. We are now at a pivotal point, and what happens next is likely to dictate the future of infectious disease control. Genomics has outlined several repeating patterns in the emergence and spread of AMR bacteria, and although we cannot stop bacterial evolution, we can try to channel it. Through coordinated efforts, intelligent surveillance, and a more fundamental understanding of AMR mechanisms, we can learn to use antimicrobials more effectively and extend their longevity.
